# MicroRNAs as Mediators of the Ageing Process

**DOI:** 10.3390/genes5030656

**Published:** 2014-08-20

**Authors:** Lorna W. Harries

**Affiliations:** RNA-Mediated Mechanisms of Disease Group, Institute of Biomedical and Clinical Sciences, University of Exeter Medical School, Barrack Road, Exeter EX2 5DW, UK; E-Mail: L.W.Harries@exeter.ac.uk; Tel.: +44-1392-406-749; Fax: +44-1392-406-767

**Keywords:** miRNA, ageing, senescence

## Abstract

Human ageing is a complex and integrated gradual deterioration of cellular processes. There are nine major hallmarks of ageing, that include changes in DNA repair and DNA damage response, telomere shortening, changes in control over the expression and regulation of genes brought about by epigenetic and mRNA processing changes, loss of protein homeostasis, altered nutrient signaling, mitochondrial dysfunction, stem cell exhaustion, premature cellular senescence and altered intracellular communication. Like practically all other cellular processes, genes associated in features of ageing are regulated by miRNAs. In this review, I will outline each of the features of ageing, together with examples of specific miRNAs that have been demonstrated to be involved in each one. This will demonstrate the interconnected nature of the regulation of transcripts involved in human ageing, and the role of miRNAs in this process. Definition of the factors involved in degeneration of organismal, tissue and cellular homeostasis may provide biomarkers for healthy ageing and increase understanding of the processes that underpin the ageing process itself.

## 1. Introduction

MicroRNAs (miRNAs) are short, non-coding RNA species that have a pivotal role in post-transcriptional regulation of gene expression. MicroRNAs associate with the RNA-induced silencing complex (RISC) and bind to the 3' untranslated region (UTR) of their target transcripts, resulting in reduction of gene expression by mRNA degradation or translational blocking [1]. The specificity of miRNA:mRNA binding is brought about by complementarity of the “seed” sequence of the miRNA (a tract of 7-8 nucleotides at the 5' end of the miRNA molecule) with a complementary sequence within the target mRNA [2]. The relatively short region of complementarity between miRNA and target results in many transcripts containing potential binding sites for a given miRNA, and a single miRNA therefore has the potential to regulate hundreds of different mRNA targets [3].

MicroRNAs are estimated to regulate as many as 60% of all human mRNAs, which represent practically all cellular and molecular functions [4]. MiRNAs are known to be key players in the regulation of transcripts involved in processes as diverse as embryonic development, differentiation, cellular proliferation, apoptosis, metabolism and adaptation to environmental stress [5]. Given the involvement of miRNA regulation in multiple cellular processes, it is unsurprising that this process plays a part in complex, multifactorial and environmentally-influenced cellular processes such as human disease and cellular and organismal ageing.

The expression profile of miRNAs may be tissue specific and labile, although some miRNAs are common to most tissues. The precise pool of miRNAs that show age-related changes is influenced by the miRNA expression profile of the particular cell type or species studied, and may also reflect the features of ageing cells which differ between tissue types or species. The heterogeneous nature of tissues, together with the known cell- and sometimes species-specificity of miRNAs may make identification of all the major players associated with ageing in humans and other species in a single tissue a difficult proposition, and also raises issues with integration of datasets. However, ageing is characterized by a well-defined set of characteristics that are shared by most tissue types and are known as “hallmarks” of ageing [6]. MicroRNAs associated with specific “hallmarks” of ageing in humans and other species may identify miRNAs that have more general importance in the ageing process distinct from those which may have a more tissue-specific role.

## 2. The Hallmarks of Ageing

**Figure 1:**
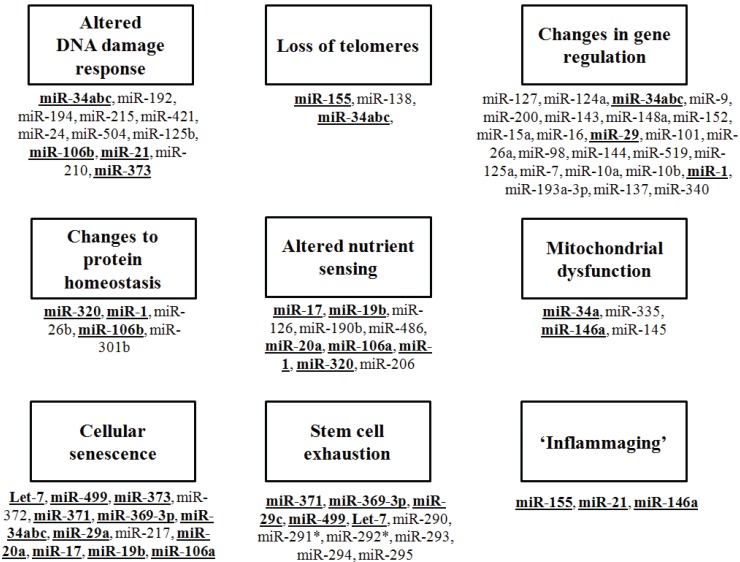
The hallmarks of eukaryotic ageing and the miRNAs that may interact with these pathways. MicroRNAs that have been to be associated with each feature are indicated. Those miRNAs that are known to be involved in more than one feature are given in bold, underlined text.

Ageing is characterized by a progressive deterioration of cellular processes in all tissues. Age-related changes are myriad, and involve almost every cellular and biological function pathway. There are several “hallmarks” of ageing, as recently described by Lopez-Otin *et al.* [6], see Figure 1. These include changes to genomic stability, brought about by accumulating DNA damage in the face of decreased DNA repair [7], the progressive shortening of telomeres as chromosomes age [8], changes in fine control of gene expression through epigenetic changes and deregulation of alternative splicing [9,10], changes to proteostatic processes such as ubiquitination, protein folding and trafficking [11], deregulation of nutrient sensing pathways such as mTOR and IGF1 signaling [12,13], mitochondrial dysfunction [14], cellular senescence [15], stem cell exhaustion [16] and alterations in intercellular communication such as heightened inflammatory response and disruption to cytokine expression [17]. Given the complexity of the changes that occur during the ageing process in man and other species, and the known role of miRNAs in mediating complex and interlinked pathways, it is unsurprising that miRNAs play a part in ageing, as in many other pivotal cellular processes. In this review, I will define some of the known pathways of ageing in man and other species, and outline the role of miRNA regulation of genes involved in each.

### 2.1. miRNAs and DNA Damage Response

The association between miRNAs and DNA damage response falls into two categories; miRNAs that are regulated by DNA damage responses, and those which target components of the DNA damage response and DNA repair pathways themselves.

#### 2.1.1. miRNAs Regulated by the DNA Damage Response

MiRNA biogenesis is a labile process, and miRNA expression is sensitive to many external stimuli. When damaged DNA is detected by the cell, double or single stranded DNA breaks produced through irradiation or as a result of base/nucleotide excision repair are detected by the Ataxia Telangiectasia Mutated (ATM) and Ataxia Telangiectasia Related (ATR) kinases. ATM and ATR activate the CHK1 and CHK2 proteins, which in turn bring about cessation of growth and cellular apoptosis through a cascade involving the p53 and RB1 proteins [18]. The p53 protein plays a pivotal part in determining global miRNA levels through its role in modulating the maturation of primary miRNA transcripts [19]. Studies in p53 deficient mouse embryonic fibroblast cells have indicated that one family of miRNAs, miR34a—miR34c has particular importance and are direct targets of p53 [20]. The action of p53 is also essential for induction of the related miRNAs miR-192, miR-194 and miR-215, which are up-regulated by genotoxic stress and are capable of inducing cell cycle arrest by causing increased expression of multiple transcripts involved in S phase and G1 and G2 checkpoints in human colon cancer samples [21] and human osteosarcoma cells [22].

#### 2.1.2. miRNAs that Regulate Components of the DNA Damage Response and DNA Repair

The DNA damage response is a complex, coordinated pathway of inter-regulated gene expression. Genes involved in multiple parts of this pathway are targeted by miRNAs. Firstly, the DNA damage transducer genes, *ATM* is itself targeted by miR-421 in Hela cells [23], whilst H2AX is regulated by miR-24 in terminally differentiated human blood cells [24]. Ectopic expression of miR-421 causes a phenotype resembling that seen in *ATM* patients characterized by cellular checkpoint changes and radiosensitivity [23], whereas miR-24 mediated suppression of *H2AX* causes sensitivity to gamma-radiation and genotoxic drugs [24]. Other components of the DNA damage response are also targeted by miRNAs; the *TP53* gene itself which encodes p53 is targeted by miR-504 and miR-125b in several cell lines [25,26] whereas the *CDKN1A*/p21 gene was demonstrated to be directly regulated by miR-106b in samples from five different solid tumours (breast, colon, kidney, gastric, and lung) when compared with non-cancer control tissue [27]. CDC25A, a G2/M checkpoint control gene is regulated by miR-21, a miRNA which has been shown to be under-expressed in subset of CDC25A over-expressing colon cancer samples [28]. As well as representing transcriptional targets of p53, miRNAs miR-34a, miR-34b and miR-34c target several key mRNAs in DNA damage response itself, including transcripts involved in cell cycle arrest in the G1-phase or cellular apoptosis such as *CDK4* and *CCNE2*. Finally, transcripts coding for proteins involved in repair of damaged DNA have also been demonstrated to be under the control of miRNAs in HeLa and MCF-7 breast cancer cells under hypoxic stress; miR-210 and mi-373 have been demonstrated to target the *RAD52* and *RAD23B* genes, which are involved in homologous recombination and nucleotide excision repair [29]. New evidence also suggests that miRNAs have a direct role in transcriptional regulation and chromatin histone modification, by miRNA-dependent recruitment of Argonaute to transcriptional start sites [30].

### 2.2. miRNAs and Telomere Attrition

Ageing in man and other species is accompanied by progressive shortening of the telomere repeats at the end of eukaryotic chromosomes [31]. Telomeres are maintained by the binding of a complex of proteins which includes the TRF1 and TRF2 proteins, the telomerase protein TERT itself and accessory factors EST1 and dysterin [32]. Several miRNAs have been linked with telomere maintenance; The *TRF1* gene is known to be a target of miR-155 [32] and *TERT* transcripts themselves are targeted by miR-138 in human anaplastic thyroid carcinoma cell lines [33]. In addition to its role in regulation of DNA damage response, the miR-34 family may also regulate telomere length; miR-34 levels were associated with telomere length in a series of gall bladder adenocarcinomas [34]. Telomere length must be carefully controlled by the cell; too few repeats and the cell will undergo premature senescence, and too many and the cell may not be able to adequately control cellular “lifespan” leading to diseases such as cancer. Telomerase expression is only reported at high levels in the embryonic state, thereafter it is suppressed [35]. It is therefore easy to see how altered expression of miRNAs during ageing could influence the expression of telomeric components and influence cellular lifespan.

### 2.3. miRNAs, Splicing and the Epigenetic Machinery

#### 2.3.1. miRNAs and DNA Methylation

There is a complex interplay between miRNA regulation and epigenetics. Epigenetic changes during human ageing are now relatively well understood [36]. Firstly, genes encoding miRNAs, like mRNAs, are regulated by epigenetic changes such as altered DNA methylation. In 2006, Saito *et al.* demonstrated that levels of miR-127 were elevated in bladder carcinoma cells cultured in the presence of the DNA methyl-transferase inhibitor 5-aza-dC [37]. Similarly, silencing of the miR-124a, miR-34, miR-9 and miR-200 gene families by DNA methylation or histone modifications have been noted in several studies [38,39,40,41]. Secondly, miRNAs are also capable of causing aberrant DNA methylation, as well as being a consequence of it. The miR-29 gene family directly targets the global DNA methyltransferases *DNMT3A* and *DNMT3B* in lung cancer cells [42], as do miR-143, miR-148a and miR-152 in in colorectal cancer, malignant cholangiocytes or hepititis B induced hepatocellular carcinoma cells [43,44,45]. Changes in *DNMT3* gene expression brought about by altered expression of any of these miRNAs could be an explanation for the changes in DNA methylation that is observed during human ageing [46].

#### 2.3.2. miRNAs and Histone Modifications

In a similar fashion to that described above, like genes encoding messenger RNAs, miRNA genes are also subject to regulation by histone modifications. Treatment of cell lines with compounds that inhibit histone deacetylases have been shown to result in altered miRNA profiles in SKBr3 breast cancer cells [47]. MicroRNAs miR-15a, miR-16 and miR-29 have been shown to demonstrate lower expression upon increased expression of *HDAC1*, *HDAC2* and *HDAC3* transcripts, which are responsible for histone modifications in chronic lymphocytic leukaemia samples [48]. It has also been demonstrated that altered miRNA expression can cause aberrant histone modifications. The *EZH2* gene is a component of the polycomb repressive complex (PRC) which acts to trimethylate H3K27 leading to silence genes [49]. Several miRNAs including miR-101, miR-26a, miR-98, miR-miR-124 and miR-144 are thought to regulate *EZH2* in various cell types including bladder transitional carcinoma, recurrent nasopharyngeal cancer samples and C2C12 mouse myoblasts [50,51,52,53,54]. Other PRC family members such as the *BMI1* and *RING2* transcripts are also subject to miRNA regulation in cancer cells *in vitro* and prostate cancer tissues [55]. Age-related alterations to the levels of miRNAs that regulate the global epigenetic machinery again, are likely to have profound effects on gene expression during ageing in man and other species.

#### 2.3.3. miRNAs and Regulation of Splicing

Alternative splicing is another mechanism that allows fine tuning of gene expression and precise control over responses to intra- and extracellular challenges. Over 95% of human genes are alternatively spliced, and a proportion of these alternative isoforms will have divergent 3' UTR sequences to their parent isoform. Alternative splicing paired with differential miRNA targeting of expressed isoforms with divergent 3' UTRs represents a potent mechanism for regulation of gene output [56]. MicroRNAs may also play a more direct role in the control of mRNA splicing. In a recent study, we identified that genes that control the splicing process were the major class of transcripts that show robust and reproducible expression changes with age in the human population [9,57]. Modification of levels of splicing regulators by the action of miRNAs is one potential explanation for this. Small RNA regulation of some of the splicing control proteins has previously been reported; for example, the *ELAV* transcript, which encodes the HuR protein, a major modulator of mRNA stability and translation in addition to mRNA splicing [58], is subject to regulation by miRNAs miR-519, miR-16 and miR-125a in a variety of cell types including cervical, ovarian and colon cancer cell lines [59,60,61]. Control of splice site usage is also regulated by two classes of regulator, the SR and HNRNP groups of proteins [62]. Several classes of mRNAs encoding SR proteins are known to be targeted by miRNAs; miR7, miR-10a, miR10b are known to target *SRSF1*, miR-193a-3p regulates *SRSF2* and miR-1 targets *SRSF9* in several cell types [63] and the genes encoding hnRNPA1 and hnRNPA0 are known to be targets of miR-124, miR-137 and miR-340 in colon cancer cells [64].

### 2.4. miRNA Control of Proteostatic Genes

Proteostasis is the biology of protein homeostasis. The proteostatic network consists of over 2000 genes involved in protein modification, folding, trafficking and degradation [65]. Deregulation of proteostasis can lead to aberrant protein folding and aggregation, typified by the beta amyloid deposition and neurofibrillary tangles that characterize Alzheimer’s Disease. Chaperone systems known to be important in ageing include *HSP40*, *HSP70*, *HSP72* and *HSP90*. MicroRNAs are known to regulate the chaperone network in several conditions including cerebral ischemia; miR-320 has been demonstrated to regulate *HSP20* transcripts during cardiac injury [66] and miR-1 is known to target *HSP72* mRNAs in cardiac ischemia [67]. Three miRNAs, miR-26b, miR-106a and miR301b, have been demonstrated to regulate *HSP70* expression, and were also shown to be significantly increased in Parkinson’s disease patients resulting in aberrant α-synuclein aggregation in Lewy bodies [68].

### 2.5. miRNAs and Nutrient Sensing Pathways

The interplay between nutrient sensing and ageing is well described in several model organisms such as rodents and *C.elegans* [12] and has also been described in humans [13]. In particular, the only pharmacological intervention known to improve lifespan, rapamycin, targets the IGF-1 and mTOR pathways [69]. Global miRNA analysis in the skeletal muscle of young and ageing humans has revealed blunted miRNA responses to resistance exercise in young, but not old skeletal muscle *in vivo*, and that in particular, miR-126 emerges as an important regulator of *IGF1* transcripts in muscle from older subjects [70]. Similar global profiling approaches have revealed other miRNAs may target the nutrient sensing pathways; levels of miR-190b were found to be elevated in samples from patients with hepatocellular carcinoma, and were demonstrated to be associated with low serum IGF1 expression and insulin resistance in these patients [71]. MicroRNA miR-1 has also been documented to directly target *IGF1* transcripts in cardiac and skeletal muscle [72], as have miR-320 and miR-206 in a rat model of myocardial infarction [73]. MicroRNAs miR-17, miR-19b, miR-20a and miR-106a have been shown to target *PTEN*, which encodes a major silencer of the AKT-mTOR pathway [74]. Alterations in the levels of these miRNA species therefore are predicted to modify the PTEN-related silencing of mTOR, with the effect of moderation of lifespan. These miRNAs also form a potential link between the DNA damage response and age-related deregulation of nutrient sensing, since p53, an important component of damage response is known to transcriptionally activate the miR17-92 cluster which encodes these miRNAs [75].

### 2.6. miRNAs Involved in Mitochondrial Dysfunction

Mitochondrial dysfunction is a feature of ageing. During cellular metabolism, the mitochondrial genome is at particular risk from the reactive products of respiration, by virtue of its proximity to their site of production. The mitochondrion is protected from the adverse effects of free radicals and other reactive species by a portfolio of genes including superoxide dismutase 2 (*SOD2*) and thioredoxin reductase 2 (*TRDX2*) genes which encode antioxidant enzymes. Two miRNAs have been reported which regulate these enzymes; miR-335 and miR-34a. Ageing renal mesangial cells demonstrate elevated levels of both miRNAs. Overexpression of both forms *in vitro* led to premature cellular senescence, whereas antisense-mediated knockdown of miR-335 and miR-34a in old cells delayed cellular senescence [76]. *SOD2* mRNAs are also targeted by miR-146a and altered expression of *SOD2* mRNAs was noted upon overexpression or inhibition of this miRNA in prostate cancer PC12 cells [77]. MicroRNA miR-145 has also been implicated in protection of cardiomyocytes against peroxide-induced apoptotic injury, by virtue of its repressive role in the regulation of *BNIP3* transcripts, which encode a component of the mitochrondrial apoptosis machinery [78].

### 2.7. miRNAs Involved in Cellular Senescence

Cellular senescence is the consequence of telomere attrition, DNA damage signaling, oxidative stress and oncogene expression. Proteins such as p16 (encoded by *CDKN2A*), p53 (encoded by *TP53*) and p21 (encoded by *CDKN1A*) are key players in this process [79]. Components of the miRNA biogenesis machinery itself have been associated with cellular senescence [80]. The processes by which miRNAs undergo global processing and maturation of miRNAs includes the *DROSHA* gene product which cleaves the pri-miRNA to yield the pre-miRNA, and the *DICER* gene product which cleaves the Pre-miRNA to yield the mature miRNA species [81]. Loss of DICER, with its resulting effect on miRNA biogenesis, was found to trigger the senescence process in embryonic fibroblasts which results from the activation of the CDKN2A-ARF and p53 components of the DNA damage checkpoint [82]. Specific miRNAs have also been associated with cellular senescence. These closely overlap with those that regulate DNA damage checkpoints, including miR-34a, miR-24 and members of the miR-106b cluster [83]. Other DNA-damage associated miRNAs have also been associated with cellular senescence; the miR17-92 cluster and its paralogues the miR-106a and miR-106b clusters have also been implicated in cell senescence in several tissues and cell types [75,84]. Other studies have also implicated other miRNAs such as miR-29 in cellular senescence in HeLa cells and in ageing muscle by virtue of their effect on the expression of c-Myb mRNAs [85,86]. Decreased expression of these miRNAs has been shown to cause increased levels of some of their target mRNAs including *CDKN1A* [87].

### 2.8. miRNAs and Stem Cell Exhaustion

Unlike differentiated cells, stem cells retain the capacity for regeneration and growth. All organs contain stem cells, but their numbers and stem-like properties gradually decline as we age. Components of the miRNA biogenesis machinery have been associated with stem cell status. Several miRNAs have been shown to target *DICER*. Loss of stemness has been associated with differential expression of several miRNAs, notably miR-371, miR-369-5p, miR-29c, miR-499 and let-7 in mesenchymal stem cells [88]. The high mobility group A2 protein, encoded by the *HMGA2* gene, is a potent modifier of chromatin structure and has also been associated with maintenance of the stem cell state. *HMG2A* transcripts are regulated by let-7 and modification of let-7 expression has been demonstrated to cause changes in stem cell capacity by regulating *HMG2A* and *CDKN2A* expression in mouse young adult stem cells [89]. 15 potential stem cell miRNAs have been identified in mouse embryonic stem cells. The miR-290, miR-291*, miR-292*, miR-293, miR-294, and miR-295 cluster, expressed in ES cells but not in differentiated mouse embryonic fibroblasts of NIH3T3 cells, are thought particularly important in maintenance of stem cell capacity [90]. The 290~295 cluster along with the miR-302~367 and miR-17~92 clusters have been reported to be pivotal in maintenance of stemness in mouse embryonic stem cells [91].

### 2.9. miRNAs and “Inflamm-ageing”

As we age, our general levels of inflammation increase, on a background of decreased immune capacity. This phenomenon is termed “inflamm-ageing”. Sensing of threat by the immune system results in activation of several signaling pathways, including the TOLL-like receptor (TLR) and the NF-κb pathways [92]. There is evidence that a relatively small number of miRNAs, termed “inflamma-miRs” are involved in this process. The inflamma-miR group includes miR-155, miR-21 and miR-146a [93]. MicroRNA miR-146 has been shown to regulate transcripts involved in both TLR and NF-κb pathways in cells involved in vascular remodeling [94]. This trio of miRNAs have been associated with several chronic, age-related diseases in man; both miR-21 and miR-146a have been shown to modulate levels of pro-inflammatory cytokines in pancreatic beta cells [95], and also to show altered expression in plasma from patients with cardiovascular disease [96] and in cerebrospinal fluid and extracellular fluid from patients with Alzheimer’s disease [97]. Up-regulation of miR-155 has also been noted in the synovial fluid of patients with rheumatoid arthritis [98].

## 3. Conclusions

MicroRNAs clearly have great importance in ageing, as in most other cellular processes. The hallmarks of ageing are known to be interconnected; for example telomere shortening is a feature of cellular senescence, are activation of the DNA damage response and altered cytokine profiles (senescent cells secrete a cocktail of cytokines termed the senescence associated secretory phenotype or SASP). Similarly, a number of miRNAs implicated in ageing are regulators of overlapping hallmarks of ageing; for example miR-34a has been implicated in mitochondrial dysfunction [76] and telomere attrition [34], miR-29 family members have been demonstrated to regulate DNA methylation genes [42] and to be linked with stem cell exhaustion [88]. MicroRNA 106 family members have been shown to regulate some DNA damage response transcripts [27] as well as some mRNAs encoding proteostatic factors [68], whereas the miR-17, miR-19b, miR-20a and miR-106a cluster that regulate cellular senescence have also been shown to regulate moderators of the mTOR nutrient sensing pathway [75]. This suggests that the features that characterize the ageing process by miRNAs may be brought about by the action of an integrated and coordinated pool of miRNAs, although the precise make-up of that pool and the relative impact of individual miRNAs within it will probably differ from tissue to tissue. The presence of miRNAs associated with ageing, and the growing recognition that miRNAs may also circulate in the blood complexed with proteins such as Argonaute or in microvesicles, raises the possibility of miRNAs as possible biomarkers for healthy ageing in the future.
